# Assessment of Biventricular Systolic and Diastolic Function Using Conventional and Strain Echocardiography in Children with Sickle Cell Disease Surviving 1-year After Hematopoietic Stem Cell Transplant

**DOI:** 10.1007/s00246-024-03646-y

**Published:** 2024-10-04

**Authors:** Jamie K. Harrington, Michael P. DiLorenzo, Monica Bhatia, Nicholas Boscamp, Usha S. Krishnan

**Affiliations:** 1 Department of Pediatrics, Children’s Hospital Los Angeles, Division of Cardiology, Keck School of Medicine of USC, Los Angeles, CA USA; 2 Department of Pediatrics, Division of Cardiology, College of Physicians and Surgeons, Columbia University, New York, NY USA; 3 Department of Pediatrics, Division of Hematology/Oncology/Stem Cell Transplantation, College of Physicians and Surgeons, Columbia University, New York, NY USA; 4 Department of Cardiology, Boston Children’s Hospital, Boston, MA USA

**Keywords:** Sickle cell disease, Pediatric, Hematopoietic stem cell transplant, Left ventricle, Systolic function, Diastolic function, Speckle-tracking echocardiography, Strain imaging

## Abstract

Hematopoietic stem cell transplant (HSCT) is a potentially curative therapy for children with sickle cell disease (SCD). The effects of HSCT on ventricular function are not well characterized in children with SCD. Echocardiograms from children with SCD who underwent HSCT between 2007 and 2017 were retrospectively analyzed before and 1-year after HSCT. Left ventricular (LV) volumes, mass, and ejection fraction were calculated by the 5/6 area*length method. LV end-diastolic and systolic dimensions, septal, and posterior wall thickness, and fractional shortening were measured by M-mode. Mitral and tricuspid inflow Dopplers (E and A waves) as well as mitral, tricuspid, and septal tissue Dopplers (E’, A’) were assessed. E/A, E’/A’ and E/E’ ratios were calculated. Biventricular strain imaging was performed using speckle-tracking echocardiography. Peak global systolic longitudinal and circumferential LV strain, and global longitudinal right ventricular strain, as well as early and late diastolic strain rate, were measured on LV apical 4-chamber, LV short-axis mid-papillary, and RV apical views, respectively. Forty-seven children (9.7 ± 5.5 years, 60% male) met inclusion criteria. Pre-HSCT, subjects had mild LV dilation with normal LV systolic function by conventional measure of ejection fraction and fractional shortening. There was a significant reduction in LV volume, mass, and ejection fraction after HSCT, but measurements remained within normal range. LV longitudinal and circumferential strain were normal pre-HSCT and showed no significant change post-HSCT. RV strain decreased after HSCT, but the absolute change was small, and mean values were normal both pre- and post-HSCT. Conventional measures of diastolic function were all normal pre-HSCT. Post-HSCT there was a reduction in select parameters, but all parameters remained within normal range. Early and late diastolic strain rate parameters showed no significant change from pre- to post-HSCT. At one-year after HSCT in children with SCD conventional measures of systolic and diastolic function are within normal limits. Except for a small decrease in RV systolic strain with values remaining within normal limits, systolic strain and diastolic strain rate values did not significantly change 1-year after HSCT.

**Supplementary Information:**

The online version contains supplementary material available at 10.1007/s00246-024-03646-y.

## Introduction

Sickle cell disease (SCD) is an autosomal recessive disorder resulting in erythrocytes which are prone to vaso-occlusion and hemolysis leading to multisystem disease beginning in childhood [1–3]. Ventricular systolic and diastolic dysfunction are known to develop in children and young adults with SCD. [4–8] The etiology of the ventricular dysfunction is multifactorial. It is related to the left ventricular (LV) dilation which occurs secondary to the hyperdynamic circulation in the setting of chronic anemia. [9–11] In addition, chronic blood transfusions can lead to iron overload within the myocardium contributing to ventricular dysfunction. [12–14] There are also repetitive miscrovascular ischemic events leading to myocardial injury and fibrosis [15].

Hematopoietic stem cell transplant (HSCT) is a therapeutic and potentially curative option for SCD. [16] Preparative regimens involve chemotherapy and/or radiation therapy, which may have cardiotoxic effects. [16–19] The effects of HSCT on ventricular function in children with SCD is not well defined. In studies involving those undergoing HSCT for malignant conditions, ventricular function can be decreased post- HSCT; however, this may be secondary to prior cardiotoxic medication exposure [17,20]. Studies assessing the effects of HSCT on cardiac function in children without an underlying malignancy, and especially in isolated populations of children with SCD, are limited.

In most reports, the majority of children with SCD initially have preserved ventricular function with increasing incidence of ventricular dysfunction with age using conventional echocardiographic assessment of ejection fraction [8]. However, strain imaging, a more sensitive tool to assess ventricular function, may unmask underlying ventricular dysfunction in children with SCD [21]. LV systolic strain has been shown to be decreased both before and after HSCT in a mixed population of children with SCD and severe acquired aplastic anemia, though has not been characterized in an isolated SCD cohort [22]. Furthermore, the diastolic strain profile has not been well assessed in a HSCT population. In the current study, we aimed to assess biventricular systolic and diastolic function in a population of children with SCD before and one-year after HSCT using conventional and strain echocardiography.

## Materials and Methods

### Study Population

We performed a retrospective cohort study of pediatric patients with SCD who underwent HSCT between 2007 and 2017. Inclusion criteria included the diagnosis of SCD status-post HSCT with echocardiograms prior to and one year following HSCT with adequate image quality for strain analysis. Studies were defined as having adequate image quality for strain analysis if the endocardial borders were well defined and the strain software was able to adequately track the endocardium throughout the cardiac cycle. Patients who underwent HSCT for other indications, had a second HSCT within one year of the first HSCT, had inadequate echocardiogram images for analysis, or died prior to 1 year follow up were excluded.

### Clinical Data

Clinical parameters collected included demographics, age at HSCT, and time from HSCT to follow up echocardiogram. The height, weight, and body surface area were recorded at each echocardiogram. SCD history, including treatment with hydroxyurea or chronic transfusions and lifetime admissions for acute chest syndrome and pain crises was recorded. Markers of hemolysis (hemoglobin, hematocrit, total bilirubin, white blood cell count, platelet count, and hemoglobin S percentage) were recorded. The HSCT conditioning regimens were stratified based on intensity (myeloablative versus non-myeloablative). Acute graft versus host disease (GVHD) prophylaxis consisted of tacrolimus with or without additional agents. The incidence of GVHD, graft failure, and second HSCT were recorded.

### Echocardiograms

Clinically indicated transthoracic echocardiograms were performed using Phillips iE33 or EPIQ machines (Philips Healthcare, Andover, MA). All studies were performed according to the American Society of Echocardiography guidelines [23]. Images were reviewed for image quality and suitability for use in the study. LV end-diastolic and end-systolic volumes (LVEDV and LVESV, respectively), ejection fraction (EF), and mass were measured using 5/6 area*length measurements [23]. End-diastolic and systolic LV dimensions, septal and posterior wall thickness, and shortening fraction (FS) were measured on available M-mode images. Conventional measures of diastolic function included spectral and tissue Doppler analysis. E and A waves and E/A ratio from mitral (MV) and tricuspid (TV) inflow Dopplers, and tissue Doppler, mitral, tricuspid and septal E’, A’ were assessed. E’/A’ and E/E’ ratios were calculated. Z-scores were calculated for ventricular volume, mass, and dimensional measurements, as well as for available diastolic function parameters based on the Boston Children’s Hospital Z-score data [24,25].

### Strain

Biventricular strain analysis was performed using two-dimensional speckle-tracking echocardiography on previously acquired images. Peak global systolic longitudinal and circumferential LV strain (%), global longitudinal right ventricular strain (%), and early and late diastolic strain rate (1/sec) were measured on LV apical 4-chamber, LV short-axis mid-papillary, and RV apical views, respectively. All strain analysis was performed using TomTec version 4.6 (TomTec Imaging Systems, Unterschleissheim, Germany). The endocardial border was manually traced at end-systole in the selected views. The software is designed to track the endocardial layer throughout the cardiac cycle using the feature tracking algorithm incorporating both speckle tracking and border detection. The quality of the tracking was ensured for each segment and adjusted as necessary. If there was inadequate tracking after three attempts at manual adjustment, the image was not analyzed. Z-scores were calculated for LV longitudinal and circumferential strain and RV strain [26].

### Statistical Analysis

Continuous variables were described using mean with standard deviation (SD) or median with interquartile range (IQR), if not normally distributed. Categorical variables were described with counts and percentages. Pre- and post-HSCT echocardiogram parameters were compared using paired Student’s t-test or Wilcoxon signed-rank test based on distribution of data. For all analyses, statistical significance was indicated by a two-sided p value < 0.05. All data analysis was performed using STATA 14.1 (StataCorp, College Station, TX).

## Results

### Baseline

Sixty-one patients with SCD underwent HSCT during the study period. Of these 47 met inclusion criteria. Ten patients were excluded as they did not have both their pre and 1-year post-HSCT echocardiograms performed at our institution, and four patients were excluded because they had a second HSCT within the first year after their initial HSCT. The average age of subjects at HSCT was 9.7 ± 5.5 years. Most subjects were male (60%), had a sickle cell genotype HbSS (77%), and were not undergoing chronic transfusions (83%). Additional demographic information is listed in Table 1.

**Table 1:** Clinical characteristics (*n* = 47)

Variable	
HSCT age (y)	9.73 ± 5.52 9.69 (4.0, 13.56)
Male, n (%)	29 (60.4%)
*Sickle genotype, n (%)*	
HgbSS	36 (77)
HgbSB^0^	6 (13)
HgbSC	4 (8)
HgbSB^+^	1 (2)
*Hydroxyurea, n (%)*	
No	30 (64)
Yes	16 (34)
Unknown	1 (2)
*Transfusion protocol, n (%)*	
No	39 (83)
Yes	7 (15)
Unknown	1 (2)
*Acute chest syndrome, n (%)*	
0	10 (21)
1	14 (30)
> 1	14 (30)
Unknown	9 (19)
*Pain crises, n (%)*	
0	7 (15)
1	4 (8)
> 1	31 (66)
Unknown	5 (11)
*HSCT conditioning, n (%)*	
Myeloablative	40 (85)
Non-Myeloablative	7 (15)
*GVHD prophylaxis, n (%)*	
Tacrolimus/Mycophenolate	37 (79)
Mofetil	6 (13)
Tacrolimus	4 (8)
*Tacrolimus/Methotrexate*	
GVHD, n (%)	11 (23)
Graft failure, n (%)	2 (4)
Second HSCT, n (%)	0 (0)

Data are expressed as mean ± standard deviation or median (IQR)

GVHD = grade versus host disease; HSCT = hematopoieitic stem cell transplantation

Pre-HSCT labs were notable for anemia (Hgb 9.3 ± 1.4) and hyperbilirubinemia (total bilirubin 2.8 ± 2.6) with an average HbS of 56.7 ± 23.8%. Following HSCT there was significant improvement in all labs (Table 2).

**Table 2:** Laboratory data

Variable	Pre-HSCT (*n* = 47)	Post-HSCT (*n* = 47)	p
Hemoglobin (g/dL)	9.27 ± 1.42 9.2 (8.2, 10.2)	12.15 ± 1.66 12.1 (11.1, 13.2)	< 0.001
Hematocrit (%)	26.68 ± 4.16 26.1 (23.5, 29.6)	35.0 ± 4.38 34.8 (32.0, 37.8)	< 0.001
Total bilirubin (mg/dL) (n = 45)	2.80 ± 2.56 1.8 (1.4, 3.2)	0.6 ± 1.08^a^ 0.4 (0.3, 0.6)	< 0.001
WBC (× 10^3^/uL)	10.9 ± 4.1 10.4 (7.8, 13.0)	7.5 ± 3.5 7.0 (5.0, 8.8)	< 0.001
Platelets (× 10^3^/uL)	380 ± 191 369.5 (235, 474)	296 ± 141 275 (194, 365)	0.0016
Hgb S (%)	56.7 ± 23.8 65.7 (40.6. 77.3)	15.3 ± 18.0 0 (0, 35.8)	< 0.001

Data are expressed as mean ± standard deviation and median (IQR)

*Hgb S* hemoglobin S percentage; *HSCT* hematopoietic stem cell transplantation; *WBC* white blood cell count

### Echocardiography

Pre-HSCT, subjects overall had normal LV systolic function (LVEF 64 0.2 ± 4.8%) with mild LV dilation (indexed LVEDV (LVEDVi) 92.4 ± 18.8 ml/m2, Z-score (+ 2.1 ± 1.5). There was no LV hypertrophy pre-HSCT (indexed LV mass 66.5 ± 16.0, Z-score + 0.7 ± 1.7). Following HSCT there was a significant reduction in LV volume (p < 0.001), LV mass (p < 0.001), and LV EF (p = 0.045), though overall values remained within normal range (Table 3). By M-mode, all LV dimensions measured within normal limits with normal LV FS both before and after HSCT (Table 3).

**Table 3:** Conventional echocardiographic data

Variable	Pre-HSCT (*n* = 47)	Post-HSCT (*n* = 47)	p
Age (y)	9.7 ± 5.6 9.6 (3.9, 13.8)	10.8 ± 5.6 10.7 (5.0, 14.8)	
BSA (m^2^)	1.08 ± 0.43 1.0 (0.65, 1.46)	1.16 ± 0.42 1.09 (0.72, 1.52)	< 0.001
Weight (kg)	33.8 ± 20.3 26.6 (16.0, 49.4)	36.8 ± 20.2 29.8 (18.1, 49.7)	< 0.001
SBP (mmHg)	110.3 ± 12.7 108 (102, 119)	106.6 ± 14.2 104 (97, 115)	0.044
Heart Rate (bpm)	90.8 ± 16.9 93 (80, 100)	87.6 ± 17.5 90 (75, 100)	0.099
5/6 area*length measurements			
LV EDVi (ml/m^2^)	92.4 ± 18.8 91.0 (79.6, 107.1)	71.1 ± 13.8 69.4 (58.8, 79.5)	< 0.001
LV EDV Z-score	2.06 ± 1.48 2.0 (1.2, 3.0)	0.08 ± 1.29 0.01 (− 0.71, 1.06)	< 0.001
LV ESVi (ml/m^2^)	33.1 ± 8.0 34.0 (26.4, 37.4)	26.6 ± 5.5 25.5 (22.0, 30.3)	< 0.001
LV ESV Z-score	1.5 ± 1.2 1.61 (0.66, 2.55)	0.35 ± 1.2 0.50 (− 0.4, 0.96)	< 0.001
LV massi (g/m^2^)	66.5 ± 16.0 64.6 (54.9, 77.0)	54.7 ± 19.3 49.3 (43.6, 62.0)	< 0.001
LV mass Z-score	0.72 ± 1.74 0.7 (− 0.19, 1.80)	− 0.83 ± 1.77 − 1.3 (− 2.0, − 0.2)	< 0.001
LV mass:volume ratio	0.73 ± 0.17 0.73 (0.65, 0.81)	0.78 ± 0.23 0.73 (0.66, 0.91)	0.22
LV EF (%)	64.2 ± 4.8 64.4 (60.3, 67.6)	62.4 ± 5.1 60.9 (58.3, 66.5)	0.045
*M-mode measurements*			
M-mode IVSD Z-score	− 0.7 ± 1.2 − 0.7 (− 1.4, − 0.1)	− 1.1 ± 1.2 − 1.2 (− 2.2, − 0.3)	0.14
M-mode IVSS Z-score	− 0.6 ± 1.4 − 0.9 (− 1.6, 0.4)	− 1.2 ± 1.4 − 1.0 (− 2.2, − 0.4)	0.02
M-mode LV EDD Z-score	1.4 ± 1.1 1.4 (0.9, 2.2)	− 0.01 ± 0.9 0.1 (− 0.5, 0.6)	< 0.001
M-mode LV ESD Z-score	0.9 ± 1.1 0.8 (0.2, 1.6)	0.1 ± 0.9 0.07 (− 0.7, 0.8)	< 0.001
M-mode LV PWDD Z-score	− 1.0 ± 1.3 − 1.2 (− 1.9, 0.0)	− 1.1 ± 1.2 − 1.4 (− 1.9, − 0.6)	0.30
M-mode LV PWSD Z-score	− 0.3 ± 3.0 − 0.6 (− 1.6, 0.2)	− 1.5 ± 1.6 − 1.6 (− 2.8, − 0.3)	0.002
M-mode FS (%)	36.6 ± 4.1 37.4 (33.6, 39.4)	35.1 ± 3.7 35.1 (32.2, 37.9)	0.02

Data are expressed as mean ± standard deviation and median (IQR)

*BSA* body surface area; *EDD* end-diastolic dimension; *EDV* end-diastolic volume; *EDVi* indexed end-diastolic volume; *EF* ejection fraction; *ESD* end-systolic dimension; *ESV*  end-systolic volume; *ESVi* indexed end-systolic volume; *FS* fractional shortening; *IVSD* interventricular septal dimension diastole; *IVSS* interventricular septal dimension systole; *LV* left ventricle; *massi* indexed mass; *PWDD* posterior wall diastolic dimension; *PWSD* posterior wall systolic dimension; *SBP* systolic blood pressure

Pre-HSCT conventional RV and LV diastolic parameters were all normal (Table 4). The MV E and E/A ratio Z-scores, as well as the TV E significantly decreased post-HSCT. There were also significant reductions in the tissue Doppler E’ Z-scores for the MV, septum, and TV, as well as the E’/A’ ratio Z-score for the MV and TV. However, overall parameters remained within normal range (Table 4).

**Table 4:** Conventional echocardiographic diastolic function assessment

Variable	Pre-HSCT	Post-HSCT	p
*Inflow Doppler*			
MV E Z-score (n = 44)	0.4 ± 1.0 0.5 (− 0.4, 1.0)	− 0.5 ± 0.9 − 0.6 (− 1.3, 0.2)	< 0.001
MV A Z-score (n = 44)	0.8 ± 1.1 0.7 (− 0.04, 1.5)	0.6 ± 0.9 0.6 (0.2, 1.3)	0.20
MV E/A Z-score (n = 44)	− 0.4 ± 0.9 − 0.5 (− 1.0, 0.1)	− 0.8 ± 0.8 − 0.9 (− 1.4, − 0.5)	< 0.001
TV E^*^ (n = 14)	56.0 ± 14.0 54 (46, 64)	52.0 ± 10.7 52.7 (45.3, 60.3)	0.03
TV A^*^ (n = 14)	39.7 ± 14.7 36.0 (29.0, 51.2)	40.2 ± 10 39.7 (34.0, 45.7)	0.32
TV E/A^*^ (n = 14)	1.5 ± 0.5 1.5 (1.2, 1.8)	1.4 ± 0.5 1.3 (1.1, 1.5)	0.50
*Tissue Doppler*			
MV E’ Z-score (n = 44)	− 0.1 ± 0.9 − 0.05 (− 0.7, 0.4)	− 0.9 ± 1.1 − 0.9 (− 1.6, − 0.2)	< 0.001
MV A’ Z-score (n = 44)	0.6 ± 1.0 0.5 (0.1, 1.2)	0.8 ± 1.0 0.6 (0.1, 1.4)	0.14
MV E’/A’ Z-score (n = 44)	− 0.5 ± 0.9 − 0.6 (− 1.1, − 0.1)	− 1.0 ± 0.7 − 1.1 (− 1.4, − 0.7)	< 0.001
MV E/E’ Z-score (n = 42)	0.3 ± 0.9 0.2 (− 0.3, 0.7)	0.06 ± 0.8 0.1 (− 0.4, 0.5)	0.19
Septum E’ Z-score (n = 43)	− 0.1 ± 1.0 − 0.2 (− 0.7, 0.4)	− 1.0 ± 1.0 − 1.1 (− 1.6, − 0.6)	< 0.001
Septum A’ Z-score (n = 43)	1.0 ± 1.0 0.8 (0.3, 1.6)	0.8 ± 1.0 0.7 (0.2, 1.5)	0.34
Septum E’/A’ Z-score (n = 43)	− 0.8 ± 0.7 − 0.8 (− 1.3, − 0.4)	− 1.1 ± 0.7 − 1.2 (− 1.6, − 0.6)	0.08
Septum E/E’ Z-score (n = 42)	0.4 ± 1.0 0.03 (− 0.4, 1.0)	0.1 ± 0.8 − 0.05 (− 0.5, 0.8)	0.27
TV E’ Z-score (n = 36)	0.3 ± 0.9 0.5 (− 0.2, 0.8)	− 0.5 ± 1.0 − 0.4 (− 1.3, 0.2)	0.005
TV A’ Z-score (n = 36)	0.6 ± 1.2 0.2 (− 0.2, 1.2)	0.8 ± 1.3 0.7 (0.2, 1.6)	0.92
TV E’/A’ Z-score (n = 36)	− 0.2 ± 0.8 − 0.4 (− 0.8, 0.5)	− 0.6 ± 1.1 − 0.8 (− 1.3, − 0.4)	0.04
TV E/E’^*^ (n = 10)	3.4 ± 0.8 3.3 (3.0, 3.9)	4.0 ± 1.3 3.6 (3.3, 4.2)	0.2

Data are expressed as mean ± standard deviation and median (IQR)

*MV* mitral valve; *TV* tricuspid valve

^*^Z-score data not available

### Strain

**Fig. 1:**
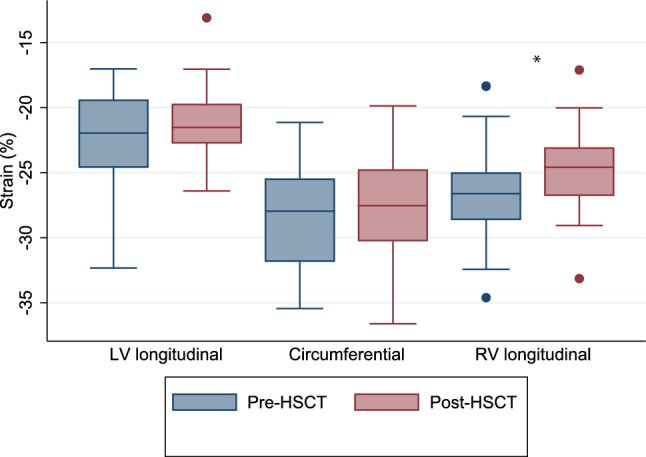
Box plots of longitudinal, circumferential, and right ventricular strain pre and post-HSCT. *p < 0.05

Strain data is shown in Table 5 and Fig. 1. Pre-HSCT there was normal LV longitudinal and circumferential peak systolic strain. There was no significant change in LV longitudinal or circumferential strain after HSCT. There was a significant reduction in RV strain from pre- to post-HSCT (− 26.6 ± 3.4 vs. − 24.7 ± 2.8%, p < 0.001), though the absolute change was small, and overall values remained within normal limits based on Z-score assessment.

**Table 5:** Strain

Variable	Pre-HSCT	Post-HSCT	p
*Peak systolic strain*			
GLS 4C (n = 47)	− 22.1 ± 3.1 − 22.0 (− 24.6, − 19.4)	− 21.1 ± 2.5 − 21.5 (− 22.8, − 19.7)	0.059
GLS 4C Z-score (n = 47)	0.55 ± 1.1 0.58 (− 0.31, 1.46)	0.85 ± 0.83 0.98 (0.06, 1.43)	0.11
GCS SAX-Mid (n = 46)	− 28.5 ± 3.9 − 28.0 (− 31.9, − 25.5)	− 27.6 ± 4.4 − 27.5 (− 30.3, − 24.8)	0.47
GCS SAX-Mid Z-score (n = 46)	0.61 ± 1.1 0.79 (− 0.42, 1.39)	0.79 ± 0.17 0.92 (0.19, 1.76)	0.28
RV GLS (n = 43) RV GLS Z-score (n = 40)	− 26.6 ± 3.4 − 26.6 (− 28.6, − 25.0) − 0.43 ± 0.92 − 0.57 (− 1.0, 0.17)	− 24.7 ± 2.8 − 24.6 (− 26.8, − 23.1) 0.11 ± 0.77 0.04 (− 0.39, 0.56)	< 0.001 < 0.001
*Diastolic strain rate*			
LSRe 4C (n = 47)	1.40 ± 0.58 1.2 (1.1, 1.5)	1.39 ± 0.55 1.2 (1, 1.5)	0.68
LSRa 4C (n = 32)	0.42 ± 0.22 0.39 (0.25, 0.57)	0.49 ± 0.28 0.40 (0.26, 0.65)	0.76
CSRe SAX-Mid (n = 46)	1.72 ± 0.60 1.65 (1.3, 1.9)	1.61 ± 0.52 1.5 (1.2, 1.9)	0.58
CSRa SAX-Mid (n = 31)	0.54 ± 0.34 0.42 (0.30, 0.66)	0.58 ± 0.38 0.43 (0.30, 0.73)	0.09
LSRe RV (n = 43)	1.38 ± 0.48 1.3 (1.04, 1.6)	1.36 ± 0.54 1.3 (1, 1.7)	0.90
LSRa RV (n = 31)	0.80 ± 0.46 0.71 (0.43, 1.22)	0.87 ± 0.41 0.86 (0.64, 1.08)	0.77

Data are expressed as mean ± standard deviation and median (IQR). Z-scores are presented when available

*4C*  apical 4-chamber view; *CSRa * late diastolic circumferential strain rate; *CSRe* early diastolic circumferential strain rate; *GCS* global circumferential strain; *GLS* global longitudinal strain; *LSRa* late diastolic longitudinal strain rate; *LSRe* early diastolic longitudinal strain rate; *SAX-Mid* short-axis view at the mid ventricle; *RV* right ventricle

**Fig. 2:**
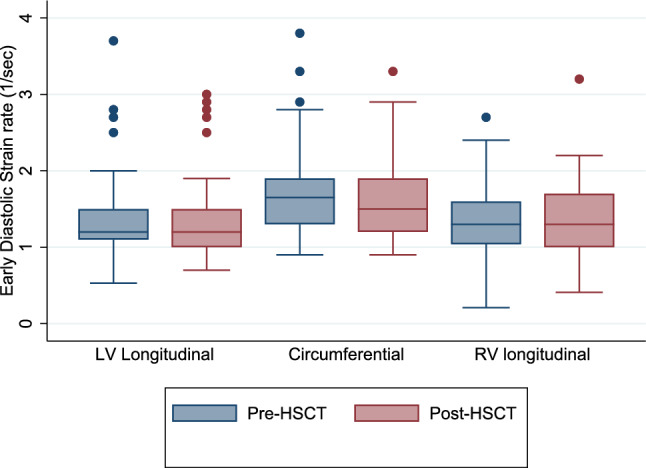
Box plots of longitudinal, circumferential, and right ventricular early diastolic strain rate

**Fig. 3:**
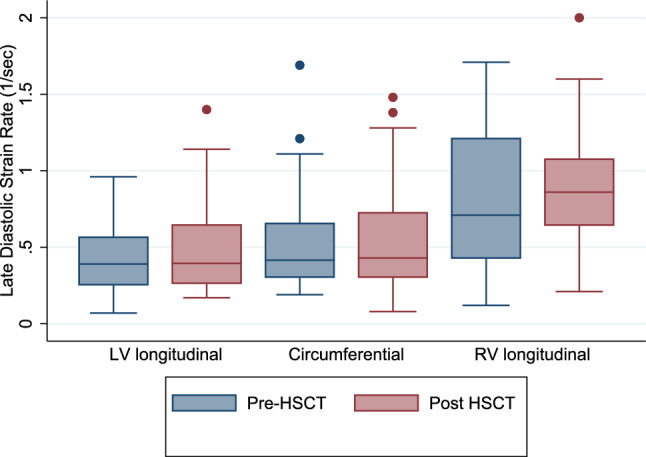
Box plots of longitudinal, circumferential, and right ventricular late diastolic strain rate

There was no significant change in LV or RV diastolic strain rates from pre- to post-HSCT (Table 5, Fig. 2 and 3).

## Discussion

This study investigated biventricular systolic and diastolic function in children with SCD before and one-year after HSCT using conventional and strain echocardiography. In our cohort, the only abnormality detected at baseline using conventional measurements was mild LV dilation with an increased LV EDVi. This finding is expected considering longstanding anemia that occurs in this population. Following HSCT, LV size normalized and there was a significant reduction in LV EF and FS, though these measurements remained within normal range. The decrease may be related to resolved anemia and a less hyperdynamic cardiac state. LV longitudinal and circumferential strain were normal pre-HSCT and showed no significant change post-HSCT. RV strain decreased after HSCT, but the absolute change was small, and overall values remained within normal limits. Conventional measures of diastolic function were normal pre- and post-HSCT, though post-HSCT there was a reduction in select parameters. Early and late diastolic strain rate parameters showed no significant change from pre- to post-HSCT.

To our knowledge, this is the largest study to date assessing ventricular function in children with SCD before and after HSCT. Most prior studies assessing ventricular function in children after HSCT have included children with a history of malignancy. Yoon et al. found evidence of subclinical LV dysfunction with decreased strain and diastolic strain rate parameters after HSCT in children with a history of acute leukemia [20]. However, the children in that study were previously treated with anthracyclines and the authors comment that the decreased function after HSCT may be related to prior anthracycline exposure with little effect of the HSCT conditioning regimen. More recently, Rotz et al. analyzed echocardiograms before and ≥ one-year after HSCT in a population of children and young adults with either malignancy or bone marrow failure syndrome [27]. They found that LV EF, as well as global longitudinal and circumferential strain were unchanged after HSCT. In this study, 46% of the cohort had prior anthracycline exposure. Similar to our study, the authors conclude that most children and young adults will not develop measurable systolic dysfunction in the first few years after HSCT.

Data on LV strain parameters in a non-malignancy HSCT population have been more mixed. The largest study to date by Covi et al [22] in 23 children with SCD or severe acquired aplastic anemia demonstrated abnormal LV longitudinal strain at baseline, with significant worsening on the first echocardiogram after HSCT. There was subsequent improvement in strain back to baseline values at one-year post-HSCT, but values were still below normal. A subgroup analysis did not show any difference in the echocardiogram findings at baseline between the SCD and aplastic anemia cohorts. However, although not significant between the small groups, the mean longitudinal strain was better in the children with SCD (-18.2%) compared to those with aplastic anemia (-15.5%). Similarly, another study which included only patients with severe aplastic anemia found that both conventional measures of systolic and diastolic function, as well as systolic strain and diastolic strain rate parameters, were significantly decreased compared to normal controls after HSCT [28]. The reasons for worse strain in these two studies may be a reflection of the inclusion of an aplastic anemia population, which may be at risk for worse longitudinal strain due to more severe anemia. Patients with aplastic anemia typically require multiple transfusions which can result in chronic iron overload in the heart with resultant cardiac dysfunction [29]. It is also possible that the SCD patients in Covi et al. had more severe disease compared to the patients in our study which may explain the abnormal longitudinal strain at baseline, or that the inclusion of patients with aplastic anemia resulted in overall abnormal longitudinal strain at baseline. A recent study by Friedman et al. showed that cardiac and pulmonary function remained stable or improved at 2-years after HSCT in children with SCD [30]. Overall, we believe that our findings support the evidence that cardiac function does not appear to be significantly worsened by HSCT in children with SCD surviving 1-year after HSCT.

RV strain has not been well assessed after HSCT in children with SCD. In our cohort, RV longitudinal strain worsened after HSCT, though overall values are still within the range of normal seen in prior reports and by Z-score analysis [26,31]. There are mixed reports on RV strain values in children with SCD. A review by Whipple et al. found that studies have reported increased, decreased, and no difference in RV strain between patients with SCD and controls. [21] A study by Whipple et al. showed that RV strain in children with SCD was better compared to normal controls and the authors hypothesized that the increased RV strain may represent a compensatory response to the hemodynamic changes seen in SCD. [32] It is possible that once the anemia is resolved after HSCT that the RV strain decreases as the compensatory response is no longer necessary. However, other studies have shown that RV strain is decreased in children with SCD.^21^ More data is needed to understand the natural history of RV strain in children with SCD, as well as the expected response after HSCT.

In the study by Covi et al., there were no significant differences in conventional measures of diastolic function one-year after HSCT compared to baseline. In our study, one-year after HSCT there was a significant decrease in select parameters of diastolic function seen predominantly in the MV and TV E and TDI E’ values and the resultant E/A and E’/A’ ratios. However, overall the conventional diastolic parameters remained within normal limits after HSCT. Robust normative data is not available for diastolic strain rate values using Tomtec; however, there was no significant change in any of the diastolic strain rate values after HSCT.

Our study is the largest to date evaluating ventricular function in an isolated population of children with SCD before and after HSCT. Overall, HSCT does not have a significant impact on ventricular function in SCD children one-year post-HSCT as compared to pre-HSCT. While a significant decrease was seen in some conventional measures of diastolic function after HSCT, values were overall well within the range of normal. In addition, the decrease in RV strain may reflect an adaptation to decreased anemia and the resultant changes in hemodynamics from a hyperdynamic to normal circulation. These findings may be particularly true in children with SCD, without a history of malignancy or more severe anemia such as seen in aplastic anemia.

This study has limitations inherent to most retrospective studies. Although this is the largest study to date assessing the impact on HSCT on ventricular function in children with SCD, the sample size is still relatively small. While findings are reassuring at one-year after HSCT, the impact of HSCT on ventricular function at longer follow-up intervals is not known. Our echo machines may have been different pre- and post-HSCT; however, as strain was all performed using the same software, this should not have resulted in significant changes in strain values. Normative data for strain and strain rate values are still being developed which may influence our assessment of normal strain parameters in these children.

In conclusion, although chemotherapy and radiation therapy used in conditioning regimens for HSCT may have an impact on cardiac function, our findings indicate that at one-year after HSCT there does not seem to be a significant negative impact on ventricular function in survivors, by conventional measures or strain imaging, in a contemporary cohort of children undergoing HSCT for SCD. This finding is reassuring and supports the evidence that HSCT prevents further cardiac damage in children with SCD. Long term studies are necessary to ensure post-HSCT ventricular failure does not occur.

## Supplementary Information

Below is the link to the electronic supplementary material.

Supplementary file1 (DOCX 16 KB)

## Data Availability

Data is provided upon request.

## Abbreviations

EDVi: Indexed end-diastolic volume
EF: Ejection fraction
FS: Fractional shortening
GVHD: Graft versus host disease
HSCT: Hematopoietic stem cell transplantationLV: left ventricular (ventricle)
MV: Mitral valve
RV: Right ventricular (ventricle)
SCD: Sickle cell disease
TV: Tricuspid valve
