# Supplementary material for: Assessment of Biventricular Systolic and Diastolic Function Using Conventional and Strain Echocardiography in Children with Sickle Cell Disease Surviving 1-year After Hematopoietic Stem Cell Transplant
Source: Pediatr Cardiol. 2024 Oct 4;46(7):1892–900. doi: 10.1007/s00246-024-03646-y (PMC11968441; doi:10.1007/s00246-024-03646-y)
Supplement: Supplementary file 1 — Supplementary file1 (DOCX 16 KB) [file 246_2024_3646_MOESM1_ESM.docx]

**Supplemental Table 1. Conventional echocardiographic diastolic function assessment (absolute measurements)**

| **Variable** | **Pre-HSCT** | **Post-HSCT** | **P** |
| --- | --- | --- | --- |
| **Inflow Doppler** |  |  |  |
|  |  |  |  |
| MV E | 98.84 ± 17.89  100.67 (87, 108.67) | 82.42 ± 15.73  80.33 (69.83, 92) | **<0.001** |
| MV A | 54.21 ± 13.72  54 (44.33, 63) | 51.84 ± 11.70  50.5 (46, 61.67) | 0.16 |
| TV E | 56.87 ± 14.08  54 (47, 65) | 51.57 ± 10.74  52.67 (44.67, 59.17) | **0.01** |
| TV A | 40.51 ± 14.79  36 (30.33, 52.33) | 40.33 ± 10.52  39.83 (34.33, 45.33) | 0.24 |
| MV E/A | 1.92 ± 0.56  1.90 (1.53, 2.19) | 1.67 ± 0.51  1.60 (1.32, 1.88) | **<0.001** |
| TV E/A | 1.52 ± 0.46  1.41 (1.19, 1.73) | 1.36 ± 0.46  1.25 (1.1, 1.46) | 0.33 |
| **Tissue Doppler** |  |  |  |
|  |  |  |  |
| MV E’ | 17.17 ± 2.9  17.67 (15, 19.33) | 15.34 ± 3.30  14.67 (13, 17.33) | **<0.001** |
| MV A’ | 6.72 ± 1.64  6.67 (6, 7.67) | 7.07 ± 1.62  6.67 (6, 8) | 0.16 |
| MV E’/A’ | 2.76 ± 1.01  2.56 (2.04, 3.11) | 2.28 ± 0.74  2.17 (1.83, 2.53) | **0.001** |
| MV E/E’ | 5.87 ± 1.53  5.63 (4.70, 6.48) | 5.48 ± 1.25  5.58 (4.62, 6.23) | 0.099 |
| Septum E’ | 12.60 ± 1.79  12.5 (11.67, 13.33) | 11.08 ± 1.92  10.83 (9.67, 12) | **<0.001** |
| Septum A’ | 7.15 ± 1.49  6.83 (6, 8) | 6.92 ± 1.52  6.83 (6, 8) | 0.21 |
| Septum E’/A’ | 1.83 ± 0.44  1.78 (1.44, 2.06) | 1.69 ± 0.48  1.60 (1.39, 1.95) | 0.13 |
| Septum E/E’ | 7.79 ± 2.08  7.61 (6.56, 8.81) | 7.46 ± 1.40  7.18 (6.71, 8.43) | 0.40 |
| TV E’ | 15.36 ± 2.71  15.67 (13.33, 16.67) | 12.98 ± 2.98  13.17 (11.17, 14.67) | **0.006** |
| TV A’ | 9.67 ± 2.79  9 (8, 11) | 10.15 ± 2.91  9.83 (8.67, 12) | 0.95 |
| TV E’/A’ | 1.7 ± 0.53  1.55 (1.4, 2.1) | 1.4 ± 0.71  1.32 (1.06, 1.56) | 0.054 |
| TV E/E’ | 3.50 ± 0.82  3.30 (2.96, 4.10) | 3.99 ± 1.31  3.55 (3.20, 4.2) | 0.37 |

Data are expressed as mean ± standard deviation and median (IQR).

MV = mitral valve; TV = tricuspid valve
